# Supplementary figures and images for: Robust 3D object watermarking scheme using shape features for copyright protection
Source: PeerJ Comput Sci. 2024 Apr 30;10:e2020. doi: 10.7717/peerj-cs.2020 (PMC11157590; doi:10.7717/peerj-cs.2020)

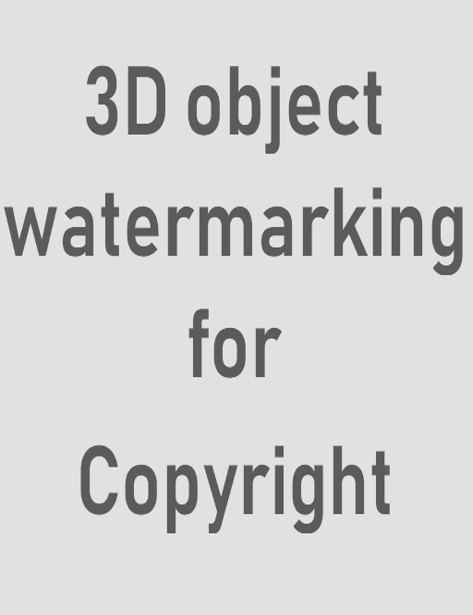

Supplement: Supplemental Information 1 [file peerj-cs-10-2020-s001.zip › Proposed method/Proposed method/Msg/Copyright615 473.png]
